# Changes in the location of cortico-muscular coherence following stroke^☆^

**DOI:** 10.1016/j.nicl.2012.11.002

**Published:** 2012-11-13

**Authors:** Holly E. Rossiter, Christiane Eaves, Emma Davis, Marie-Hélène Boudrias, Chang-hyun Park, Simon Farmer, Gareth Barnes, Vladimir Litvak, Nick S. Ward

**Affiliations:** a Sobell Department of Motor Neuroscience and Movement Disorders, UCL Institute of Neurology, London, UK; b Wellcome Trust Centre for Neuroimaging, UCL Institute of Neurology, London, UK

**Keywords:** CMC, cortico-muscular coherence, MEG, magnetoencephalography, EMG, electromyography, fMRI, functional magnetic resonance imaging, TMS, transcranial magnetic stimulation, M1, primary motor cortex, PCA, principal component analysis, MVC, maximum voluntary contraction, DICS, dynamic imaging of coherent sources, Magnetoencephalography, Cortico-muscular coherence, Stroke recovery, Motor, Brain

## Abstract

Stroke results in reorganization of residual brain networks. The functional role of brain regions within these networks remains unclear, particularly those in the contralesional hemisphere. We studied 25 stroke patients with a range of motor impairment and 23 healthy age-matched controls using magnetoencephalography (MEG) and electromyography (EMG) to measure oscillatory signals from the brain and affected muscles simultaneously during a simple isometric hand grip, from which cortico-muscular coherence (CMC) was calculated. Peaks of cortico-muscular coherence in both the beta and gamma bands were found in the contralateral sensorimotor cortex in all healthy controls, but were more widespread in stroke patients, including some peaks found in the contralesional hemisphere (7 patients for beta coherence and 5 for gamma coherence). Neither the coherence value nor the distance of the coherence peak from the mean of controls correlated with impairment. Peak CMC in the contralesional hemisphere was found not only in some highly impaired patients, but also in some patients with good functional recovery. Our results provide evidence that a wide range of cortical brain regions, including some in the contralesional hemisphere, may have influence over EMG activity in the affected muscles after stroke thereby supporting functional recovery.

## Highlights

► We examined cortico-muscular coherence location in stroke patients and controls. ► The location of peak coherence was more widely distributed in stroke patients. ► In some patients, peak coherence was found in the contralesional hemisphere. ► The location of coherence in patients did not correlate with impairment. ► Contralesional hemisphere can support functional motor recovery after stroke.

## 1 Introduction

After stroke, both functional magnetic resonance imaging (fMRI) and electroencephalography (EEG) studies have demonstrated alterations in brain activity during movement of the affected hand, particularly in the contralesional hemisphere (Ward et al., 2003; Serrien et al., 2004; Gerloff et al., 2006a; Cramer, 2008). However, the most widespread changes are seen in those patients with more impairment and it is still unclear whether new task related brain activity, particularly within the contralesional hemisphere, is supporting or hindering recovered motor function. Evidence of the former is provided by studies in which single pulse transcranial magnetic stimulation (TMS) to dorsal premotor cortices in either hemisphere disrupted motor performance in chronic stroke patients but not in control subjects (Johansen-Berg et al., 2002; Fridman et al., 2004; Lotze et al., 2006). Evidence of the latter comes from the finding that contralesional primary motor cortex (M1) in chronic stroke patients maintains an inhibitory influence over ipsilesional M1 during both movement preparation and execution (Murase et al., 2004). This finding in particular has led to small clinical studies using cortical stimulation to suppress activity in the contralesional hemisphere in order to enhance training effects on upper limb function, via interhemispheric effects on ipsilesional M1 (Stagg et al., 2012; Liepert et al., 2007; Talelli et al., 2012; Seniów et al., 2012). The results have been mixed, raising the possibility that contralesional cortical motor regions actually contribute to motor recovery in some but not all stroke patients.

One way of addressing this question directly is to determine which cortical regions have the most direct influence over muscles in the affected limb. Oscillatory signals from the brain and affected muscles can be measured simultaneously during task performance using magnetoencephalography (MEG) and electromyography (EMG) respectively, and coherence between the two can be calculated (Conway et al., 1995; Halliday et al., 1998). Cortico-muscular coherence (CMC) is detected most prominently in beta (15–30 Hz) and gamma (30–80 Hz) frequency bands. Beta band coherence is highest in tasks requiring maintenance of a posture (Kilner et al., 2000; Baker et al., 1997) whereas gamma band coherence increases with increasing muscle contraction strength and dynamic movements (Brown et al., 1998; Omlor et al., 2007). Beta band CMC has previously been found to reflect efferent drive from contralateral M1 to the muscle (Gerloff et al., 2006b; Braun et al., 2007). CMC therefore provides a non-invasive means of assessing the brain areas which are interacting with the muscle. Furthermore, MEG provides a method of assessing post-stroke brain activity which is not influenced by potential disturbances in neurovascular coupling that can affect blood oxygen level dependent signal used by fMRI.

Here, we performed simultaneous MEG–EMG recordings during a simple isometric hand grip to address the hypothesis that locations of peak coherence in both the beta and gamma bands would be more widely distributed in stroke patients than in healthy controls. In particular, we were interested to see whether the peak coherence would be present in the contralesional hemisphere and whether the location of this CMC was related to the level of impairment in patients.

## 2 Materials and methods

### 2.1 Subjects

Twenty-five stroke patients (mean age 52 ± 14 years, range 19–81 years; 19 male, 3 left-handed, 13 dominant hand affected) and twenty-three healthy controls (mean age 50 ± 20 years, range 23–77 years; 11 male, 2 left-handed) participated. All patients suffered from first-ever stroke and weakness of at least wrist and finger extensors and hand interossei and were not suffering from any other neurological disorder. A full written consent was obtained from all subjects in accordance with the Declaration of Helsinki. The study was approved by the Joint Ethics Committee of the Institute of Neurology, UCL and National Hospital for Neurology and Neurosurgery, UCL Hospitals NHS Foundation Trust, London.

### 2.2 Behavioural testing

All patients were scored on the following outcome measures; 1) action research arm test, 2) grip strength, 3) nine hole peg test, and 4) box and block timed test. A principal component analysis (PCA) was performed on these scores to take account of floor and ceiling effects and the first component was used as a single impairment score per patient.

### 2.3 Motor task/experimental paradigm

The subjects performed visually cued isometric hand grips with a manipulandum during MEG recording. Prior to scanning, maximum voluntary contraction (MVC) was recorded for each subject. Patients used their affected hand and controls were scanned using each hand in separate blocks. For each hand, 2 × 8 min blocks of 60 trials were performed. The cue to perform a hand grip was the appearance of a ‘force thermometer’ on the screen which provided continuous visual feedback about the force exerted. The target force was set between 15 and 30% of their MVC and displayed visually. Each grip was sustained for 3 s with an interstimulus interval between 3 and 7 s. A manipulandum was placed in the inactive hand to check for mirror movements.

### 2.4 MEG recording

MEG signals were measured continuously at 600 Hz during the task using a whole-head CTF Omega 275 MEG system (CTF, Vancouver, Canada). Head localization was monitored continuously during the recordings in order to check for excessive movement. The MEG data were pre-processed offline using SPM8 (Wellcome Trust Centre for Neuroimaging, www.fil.ion.ucl.ac.uk/spm) (Litvak et al., 2011). Data were down-sampled to 300 Hz and were filtered from 5–100 Hz. Data were epoched from − 3 to + 3 s where time 0 indicated onset of the visual cue. Data with large eye blinks or other artifacts were excluded.

### 2.5 EMG recording

Bipolar surface electrodes were used to record EMG from flexors and extensors of the forearm involved in grip during the task. EMG was recorded as part of the MEG dataset and so had the same pre-processing parameters. The EMG channel was rectified (Myers et al., 2003). The force output from the two manipulandi was also recorded as part of the MEG dataset in order to check that the task was being performed accurately.

### 2.6 Structural MRI recording

A 3T Siemens Trio scanner (Siemens, Erlangen, Germany) was used to acquire high resolution T1-weighted anatomical images (1.3 × 1.3 × 1.3 mm voxels); 176 partitions, (FoV = 256 × 240, TE = 2.48 ms, TR = 7.92 ms, FA = 16°). Structural MRIs could not be obtained in one of the patients and three of the controls due to MRI contraindications.

### 2.7 Data processing and analysis

For control subjects, we used data acquired during either left or right hand grip in order to match the patient group for both age and dominance of the hand used to perform the task. For further analysis, scans acquired during right hand use were flipped about the sagittal plane, so that the right hemisphere was therefore contralateral to the moving hand in all subjects. In the case of the patient group, the right hemisphere was always the ipsilesional side and all affected hands were on the left side. This enabled comparisons across subjects.

Lead fields were computed using a single-shell head model (Nolte, 2003) based on an inner skull mesh derived by inverse-normalizing a canonical mesh to the subject's individual MRI image (Mattout et al., 2007). For subjects without individual MRI the canonical mesh was affine-transformed to fit their MEG fiducials. Coregistration between the MRI and MEG coordinate systems used three fiducial points: nasion, left and right pre-auricular. While acquiring the structural MRI, fiducial points were marked with vitamin-E capsules in order to coregister with the MEG fiducials.

The beamforming method is based on the linear projection of sensor data using a spatial filter computed from the lead field of the source of interest and either the data covariance (time domain) (Van Veen et al., 1997) or cross-spectral density matrix (frequency domain) (Gross et al., 2001). Dynamic imaging of coherent source (DICS) analysis was used to calculate the coherence between the MEG sensors and EMG signal in both beta (15–30 Hz) and gamma (30–80 Hz) frequency bands in the time window 0.5 s to 3 s after the visual cue. The location of peak coherence was determined after the source localization results were thresholded to one standard deviation (computed across voxels) above the mean.

The coherence values were computed on a 3D grid in Montreal Neurological Institute space with spacing of 5 mm bounded by the inner skull surface (regularization = 1%). Values at the grid points were then linearly interpolated to produce volumetric images with 2 mm resolution.

The primary interest was the location of peak CMC in stroke patients compared to controls. To investigate this, we performed Hotelling's T-squared test (Hotelling, 1931) to examine (multivariate) differences in peak location between patient and control groups. We used the same test to identify individual patients whose peak location significantly differed from that of the control group. In addition, the distance of each patient's coherence peak from the mean control group coordinate was calculated.

**Fig. 1:**
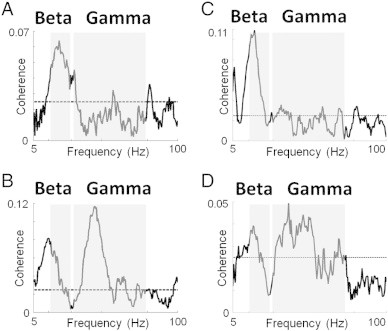
Coherence plots of control participants and stroke patients. Coherence plots between peak CMC location and EMG in representative control participants and stroke patients (95% confidence interval shown as dotted line). A) Control subject with a clear beta coherence peak but no clear gamma peak, B) Control subject with coherence in both the beta and gamma range, C) Stroke patient with a clear beta coherence peak but no clear gamma peak, and D) Stroke patient with coherence in both the beta and gamma range (patients number 7 and 14 in the table respectively).

The source signal was extracted from the peak beta and gamma coherence coordinates using Linearly Constrained Maximal Variance (LCMV) beamformer (Van Veen et al., 1997). The source orientation was in the direction yielding maximal signal variance. A coherence plot was generated between the MEG source signal and EMG channel order to see the coherence values at that location. The coherence value is bounded between 0 and 1. All coherence spectra were thresholded at the 95% confidence interval (Rosenberg et al., 1989) (Fig. 1). Participants were included in further analysis if they had CMC above the 95% confidence interval threshold.

## 3 Results

**Fig. 2:**
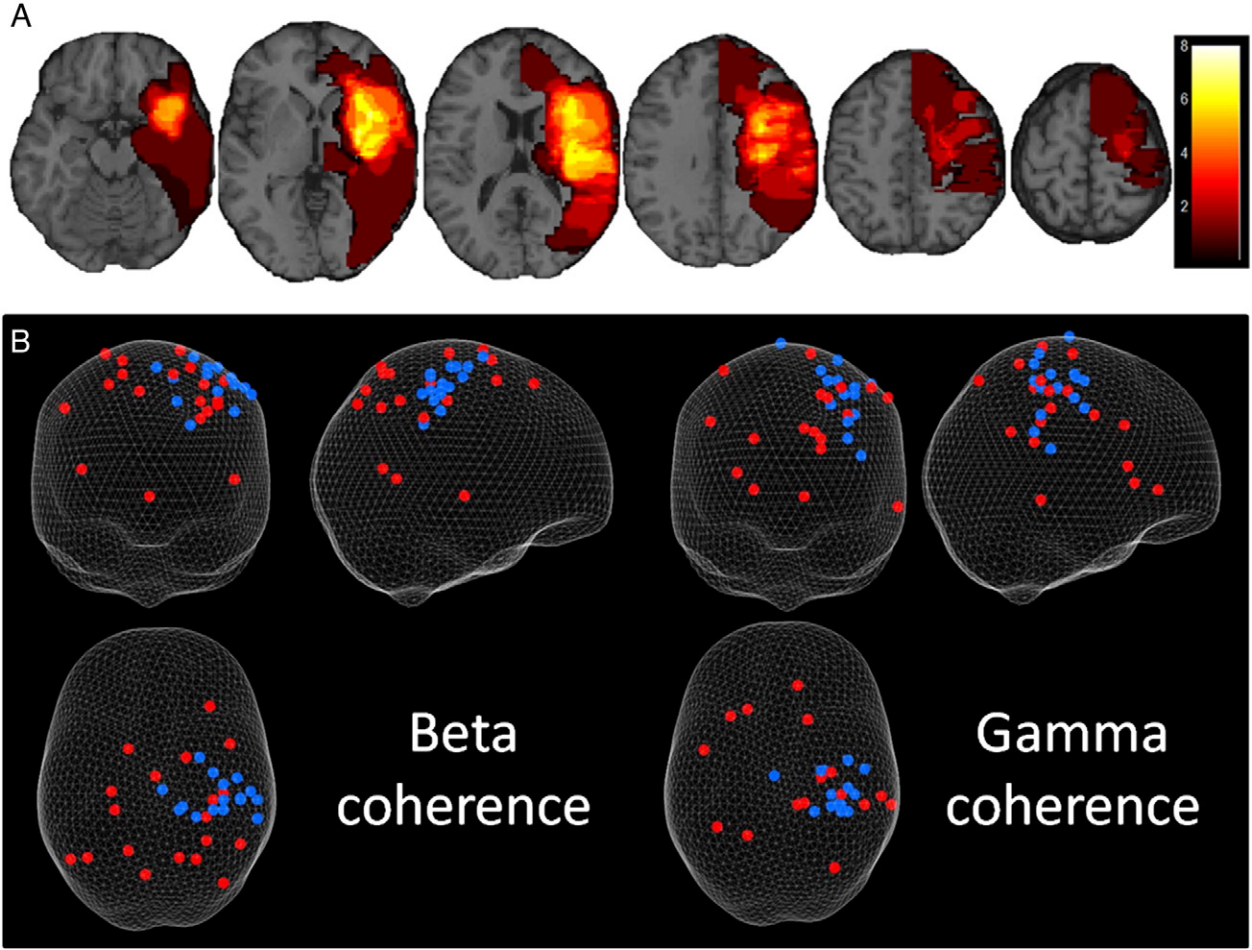
Lesion overlap and CMC coordinates on glass brain. A) Lesion overlap of stroke patients from axial slices on a template brain demonstrating the variety in cortical and subcortical damage across the group. Scale indicates number of patients overlapping. B) 3D plot of peak coherence coordinates for beta (left) and gamma (right) (grip performed with left hand — right hand grips were flipped in the sagittal plane so that all data could be included on the same plot). Control subjects are shown in blue and patients are shown in red. Results are displayed on a ‘glass brain’ and shown from behind (top left), from the right side (top right) and from above (bottom left). These peaks of CMC were calculated using a DICS beamformer.

All subjects were able to perform the grip task adequately and the target accuracy did not vary between the two groups (the average force was 18 ± 1.5% of MVC for controls and 16 ± 2% for patients). Patient baseline characteristics and infarct locations are shown in Table 1 and Fig. 2A respectively, all but two patients (patients 12 and 21 in Table 1) had sparing of the hand area of the primary motor cortex. The first PCA component explained 85% of the variance of all 4 outcome scores and so was used as the representative behavioural score.

In both groups, peaks of CMC were found in beta and/or gamma bands (Fig. 1), but significant peaks were absent in some subjects. In total the number of datasets included for further analysis was 19 patients and 16 controls for beta and 19 patients and 15 controls for gamma.

Our principal question was whether the peak of either the beta or gamma CMC would be different in patients compared to controls. In healthy controls, peak CMC was located in contralateral sensorimotor cortex for both the beta and gamma bands but in stroke patients, these peaks were more widely distributed, including 7 within the contralesional hemisphere for beta CMC and 5 for gamma CMC (Fig. 2B).

There was a significant difference between the location of peak CMC for the patient group compared to the control group (average coordinate of controls for beta CMC: 39, − 28, 61, and for gamma CMC: 32, − 27, 57; Hotelling's T-squared test, p = 0.002 for beta coherence and p = 0.016 for gamma coherence). 15 of the 19 patients' beta CMC and 13 of the 19 patients' gamma CMC peak locations were found to be significantly different (p < 0.05), from those of the control subjects (by chance one would expect 1 out of 20). Patients whose peak CMC was not different to that of the control subjects will be referred to as ‘normal’ and those which were different as ‘distant’.

We examined whether the location of the peak CMC was related to the degree of motor impairment. No correlation was found between the distance from average location in controls and impairment (for beta CMC, r^2^ = 0.04, p = 0.42, for gamma CMC, r^2^ = 0.17, p = 0.20). Because we cannot assume a linear relationship between impairment and location of peak CMC, we also tested whether there was a difference in impairment between those patients with ‘normal’ peak CMC location and those with ‘distant’ peak CMC, but found no differences (2-sample *t*-test, p = 0.1 for beta coherence and p = 0.2 for gamma coherence), Although not significant, the direction of the trends was for those with more distant CMC peaks to be more impaired.

Comparing the coherence values between patients and controls revealed no significant difference in the beta band (p = 0.72) but the gamma coherence value was significantly lower in patients than controls (p = 0.03). No correlation was found between coherence value and impairment score, although a trend was seen in the gamma band suggesting that coherence value was generally lower in more impaired patients (for beta CMC, r^2^ = 0.11, p = 0.15, for gamma CMC, r^2^ = 0.17, p = 0.08).

Coherence value in the patient group was found to correlate negatively with the distance of the peak CMC from controls for both beta (r^2^ = 0.22, p = 0.04) and gamma bands (r^2^ = 0.29, p = 0.02), with increasing distance from controls associated with lower coherence values.

There was no significant difference in peak frequency between patients and controls (p = 0.47 for beta, p = 0.72 for gamma) and no significant correlation between either beta (r^2^ = 0.01, p = 0.67) or gamma (r^2^ = 0.03, p = 0.50) peak frequency and impairment.

Some patients were found to have either (i) predominant beta coherence, (ii) predominant gamma coherence or (iii) similar levels of beta and gamma coherence (i.e. a difference in coherence value of < 0.1 between beta and gamma). We therefore compared the ages and behavioural scores of these 3 different patient groups but no significant differences were found between them in terms of age or behavioural score (p > 0.05).

As there was a variation in length of time after stroke across the patient group, an additional analysis was performed to look for significant differences between the early (stroke less than 3 months ago) and late (stroke greater than 3 months ago) patients. The behavioural scores, coherence value and distance from controls were compared between the two groups. No significant difference was found in any of these parameters. The coherence value and distance also did not correlate with time after stroke across the whole patient group. Lastly, no significant correlation was found between age and coherence value or behavioural score in healthy controls.

## 4 Discussion

In this study, simultaneous recordings were measured from the cortex and forearm muscle during isometric handgrip to find the cortical location that was most coherent with the active muscle. The results provide evidence that a wide range of cortical regions are able to influence muscle activity and are involved in supporting recovered hand function after stroke. No significant correlation was found between the coherence value or the distance of the coherence peak coordinate from controls and level of impairment. Neither were there differences in impairment levels when simply comparing those patients with ‘normal’ peak CMC location and those with ‘distant’. Previous fMRI studies have tended to show that increased task-related contralesional activity is more likely to be seen in patients with greater impairment but this was not the case in our patients with contralesional CMC. Overall, these results suggest that in some patients, contralesional hemisphere can act as a source of coherent descending cortical drive to functionally relevant muscles after stroke. In studying cortico-cortical connections, Gerloff et al. (2006a) found increased activity in the contralesional hemisphere using EEG, similarly our study found an increased involvement of the contralesional hemisphere in a number of patients. Overall these studies point to the potential influence that the contralesional hemisphere might have over motor control after stroke in some patients. Note, that our findings do not preclude continued inhibitory drive from contralesional to ipsilesional M1 as described by Murase et al. (2004), but suggest that their finding does not necessarily dominate cortical motor interactions after stroke.

The reason for such shifts in the beta and gamma CMC is not clear. In our data, those patients with contralesional CMC did not differ significantly from those with ‘normal’ peak CMC location in terms of age, time after stroke or level of impairment. Of the patients with contralesional beta and/or gamma CMC, 6 had large cortical infarcts and 2 had small subcortical infarcts. Of those patients with ‘normal’ ipsilesional beta and/or gamma CMC, there were 5 cortical and 4 subcortical infarcts. The two patients with damaged M1 had peak CMC ‘distant’ from control subjects' CMC but were not contralesional, and so we could not demonstrate consistent lesion characteristics in those with contralesional CMC. Intuitively, it would seem that the anatomy of the infarct might determine the CMC location, but from our data this does not seem to be the case. Further investigation into the cause of shifts in peak CMC is needed, in particular, longitudinal studies would be useful to investigate dynamic changes in CMC location.

The presence of ipsilateral MEPs when stimulating contralesional M1 with TMS is not consistently found and when it is seen, it is usually only seen in patients with greater impairment (Gerloff et al., 2006a; Turton et al., 1996; Netz et al., 1997). Braun et al. (2007) found neither ipsilateral MEPs using TMS in the 9 patients they studied nor CMC in contralesional M1. Hansen and Nielsen (2004) suggested that CMC and TMS measurements reflect properties in the same pathways. In healthy controls, they found that TMS over the M1 leg area increased beta CMC and stimulation of the motor nerves in the ankle muscles suppressed it. Recent primate work suggests that contralesional areas are unlikely to be connected to ipsilateral spinal cord motorneurons via fast fibres (Soteropoulos et al., 2011; Zaaimi et al., 2012). It appears more likely, at least from an anatomical perspective, that pathways such as reticulospinal (Zaaimi et al., 2012), rubrospinal (Belhaj-Saïf and Cheney, 2000) or propriospinal (Giboin et al., 2012) tracts might convey ipsilateral motor signals, although this is still relatively unclear in humans. These pathways are more likely to originate from medial or lateral premotor cortical regions in both hemispheres (23% of supplementary motor area terminations were ipsilateral and these were located mainly in laminae VII and VIII in primates (Dum and Strick, 1996)). This appears to be the most plausible explanation to date for widespread and bilateral motor task-related brain activity after stroke.

A number of studies using EEG or MEG have examined CMC after stroke (Braun et al., 2007; Mima et al., 2001). Mima et al. (2001) found (at sensor level) that CMC shifted anteriorly and/or medially away from ipsilesional M1. Braun et al. (2007) found a positive correlation between CMC magnitude and impairment. Most of these studies had small sample sizes, performed no source reconstruction and used a very specific group of, often well recovered, patients. Our study is novel in that patients with a wide variety of impairment were assessed and source level analysis was explored. Our data cannot answer how signal from such a wide variety of cortical regions can directly influence muscle activity, only that CMC is present in areas outside of ipsilesional M1. MEG spatial resolution is inferior to that of fMRI and so we are unable to state with confidence the exact gyri/sulci that are coherent with the active muscle. Nevertheless, source localization techniques such as the DICS beamformer (Gross et al., 2001) used in this study offer much more information about source location than previous sensor level work on coherence using EEG or MEG and our data at least confirm the presence of non-M1 and contralesional sources of CMC in some patients.

In many studies involving patient groups there is a concern about lower signal to noise ratio (SNR) compared to controls. However, in our dataset, similar numbers of patients and controls were excluded on the basis that no coherence peaks could be found. Still, it is important to consider whether the simplest explanation of our findings might be that the peak location in patients was more variable because of lower SNR. This could indeed partly explain the findings in the gamma band for which the magnitude of coherence was significantly lower than that in the controls. However we found no such difference in the beta band. In addition, we found no difference in coherence magnitude between those patients with ‘normal’ peak CMC location and those with ‘distant’.

In this study, a number of patients and controls lacked significant beta or gamma CMC. In those patients without significant coherence, there was a range of impairments (grip strength range 13%–108% of unaffected hand), and infarct locations (3 at the level of the internal capsule and one superior MCA territory). Furthermore, in all subjects with no measurable CMC, there was a range of ages (30–56 yrs in patients, 24–74 yrs in controls).

Interestingly, two of the patients studied showed minor mirror movements in the unaffected hand. As cortico-muscular coherence measured here used EMG from the affected hand only, the findings are unlikely to have been affected by these movements. Furthermore, those with mirror movements did not have coherence peaks in the contralesional hemisphere and those with peaks in contralesional hemisphere did not have mirror movements. Therefore, the presence of mirror movements cannot explain the contralesional coherence seen in this study.

Gamma coherence value was found to be significantly lower in patients than controls, whereas no significant difference was found in beta CMC value. Patients with more impairment had a lower gamma coherence value, although this represented a non-significant trend. Fang et al. (2009) found a similar difference between groups in both beta (20–30 Hz) and low gamma (30–40 Hz) coherence, although the difference in beta band was smaller. The reason for this difference in gamma but not in beta in both studies is not clear at the moment but could be related to the way patients perform the dynamic phase of the grip task. Gamma oscillations have been linked to the dynamic phase of movements (Omlor et al., 2007), attention (Engel et al., 2001) and also more broadly to perceptual binding (Tallon-Baudry and Bertrand, 1999), the decrease in the coherence value of gamma in patients may relate to weaker coupling between the cortex and muscle which may have arisen from cortical changes to the network due to the stroke (Fang et al., 2009), however more research needs to be done to confirm this relationship.

In summary, we provide direct evidence that brain regions in the contralesional hemisphere are involved in activity in the affected muscles after stroke thereby supporting recovered function. Our results also highlight the importance of understanding the variability in brain reorganization across stroke patients. Specifically, they suggest that the contralesional hemisphere should not necessarily be viewed as hindering recovery of motor function in all cases. This finding has important implications for how patients are stratified in rehabilitation treatment studies that aim to modulate task related hemisphere balance, particularly those involving cortical stimulation techniques, and could go some way to explain the variability of results in small scale studies using these techniques.

## Role of the funding source

This research was supported by the European Commission under the 7th Framework Program — HEALTH — Collaborative Project Plasticise (contract no. 223524) www.plasticise.eu (Dr Rossiter), the Wellcome Trust (Dr Park, Dr Ward) and the Canadian Institutes of Health Research (Dr Boudrias).

## Figures and Tables

**Table 1:** Patient demographics and behavioural scores (ACA = anterior cerebral artery, MCA = middle cerebral artery, ARAT = action research arm test, NHPT = nine hole peg test, BB = box and blocks test, asterisk represents patients who had damage to the hand region of M1). All scores (except ARAT which is scored out of 57) were reported as a percentage, the score for the affected hand was divided by the score for the unaffected hand. Patients 22–25 were excluded due to artifacts or lack of coherence. The bottom row contains the mean value ± standard deviation for the age and behavioural scores.

Patient	Gender	Age	Affected hand	Location of lesion	Months after stroke	ARAT	Grip strength (%)	NHPT (%)	BB (%)
1	Male	51	Non-dominant	Inferior MCA territory	6.8	57	80	77	92
2	Male	45	Dominant	Corona radiata/internal capsule	72	57	79	102	80
3	Male	53	Non-dominant	Posterior MCA territory	4.1	20	7	0	18
4	Female	62	Non-dominant	Corona radiata/internal capsule	7	57	60	94	86
5	Male	56	Non-dominant	Basal ganglia	1.6	57	40	108	43
6	Male	66	Non-dominant	Inferior MCA territory	84.4	50	68	14	65
7	Male	39	Non-dominant	Anterior MCA territory	16.3	1	31	0	0
8	Male	70	Dominant	Corona radiata/internal capsule	81	30	18	2	11
9	Male	54	Dominant	Corona radiata/internal capsule	105.2	56	95	50	77
10	Female	30	Dominant	Corona radiata/internal capsule	1.2	57	108	107	100
11	Male	64	Dominant	Anterior MCA territory	76	54	61	8	33
12*	Male	55	Non-dominant	Anterior MCA territory	207.9	57	79	92	86
13	Female	63	Dominant	Inferior MCA territory	0.9	57	105	100	102
14	Female	55	Non-dominant	Thalamus	2.5	49	56	50	46
15	Male	54	Dominant	Inferior MCA territory	2.5	57	93	105	88
16	Female	19	Dominant	Basal ganglia	7.3	57	112	103	95
17	Male	51	Dominant	Anterior MCA territory	21.3	23	26	0	7
18	Male	48	Non-dominant	Posterior MCA territory	1	57	43	70	88
19	Male	57	Non-dominant	Ventrolateral cerebellum	39.8	57	78	36	53
20	Male	59	Dominant	Anterior choroidal artery territory	7.3	57	110	93	93
21*	Male	37	Non-dominant	Superior MCA territory	2	0	13	0	0
22	Male	28	Dominant	Posterior MCA territory	4	28	54	0	18
23	Female	81	Dominant	Basal ganglia	3	24	35	0	16
24	Male	52	Dominant	Inferior MCA territory	35	57	73	34	69
25	Male	54	Non-dominant	Inferior MCA territory	106	57	66	68	76
Mean		52 ± 14			36 ± 51	45 ± 19	64 ± 31	53 ± 43	58 ± 35
